# C-753-09. The Future of Bio-Printing Skin: Pre-clinical and Clinical Outcomes of 3D Printing Skin In-situ

**DOI:** 10.1093/jbcr/irag033.580

**Published:** 2026-04-06

**Authors:** Jo Maitz, Alexandra Boyling, Lillian Hou, Heather Campbell, Yi Pei, Zack Artist, Jason Leavens, Peter Maitz

**Affiliations:** Burns Unit, Concord Repatriation General Hospital, Concord, NSW; Anzac Research Institute, Concord Repatriation General Hospital, Concord, NSW; Anzac Research Institute, Concord Repatriation General Hospital, Concord, NSW; Anzac Research Institute, Concord Repatriation General Hospital, Concord, NSW; Inventia Life Science, Alexandria, NSW; Inventia Life Science, Alexandria, NSW; Inventia Life Science, Alexandria, NSW; Burns Unit, Concord Repatriation General Hospital, Concord, NSW

## Abstract

**Background/Objective:**

Skin tissue engineered solutions have been adopted into clinical practice to treat all types of wounds ranging from burn injuries to chronic wounds. However, many current cell-based systems have limitations including cellular run-off and complex culturing methods. Due to these limitations, cells and their delivery systems are often adjunct treatment modalities in the management of burns. The use of highly advanced delivery systems to build constructs that represent the native human skin are at the forefront of research; we evaluate the use of a robotic 3D bio-printer in-situ to promote tissue regeneration and repair in an animal model and a first in human clinical trial.

**Methods:**

A surgical robot capable of 3D bio-printing in-situ was used to produce a cell-based construct directly into a wound, reconstructing the skin in all layers in-situ. We assessed the safety, delivery and efficacy of different autologous cell types derived from split thickness skin grafts on wounds generated in a porcine model and in a first-in-human clinical trial.

**Results:**

The results demonstrated an increase in wound closure rate of 3D printed skin compared to healing by secondary intention in the porcine model. The outcomes from this study have enabled the translation to a first in human clinical trial; a safety study to print an epidermal construct comprised of autologous keratinocytes within a 3D printed biomaterial matrix into a surgically generated wound. We present new findings of a recent analysis of the results from the clinical trial that have not been presented or published to date.

**Conclusions:**

The results from this pre-clinical and clinical study demonstrated its safety and efficacy in treating controlled and non-complicated wounds. The next phase of clinical trial is currently being prepared and will advance to partial thickness burns and full-thickness wounds. The use of this delivery system in tissue regeneration is a promising step in the advances for skin tissue engineering.

**Applicability of Research to Practice:**

This work demonstrates a clear and direct pathway from laboratory innovation to clinical implementation. By validating a robotic in-situ 3D bioprinting system in both a porcine model and a first-in-human trial, the research provides clinicians with a feasible, safe, and scalable method for delivering autologous skin constructs at the point of care. The technology offers practical advantages over conventional cell-based therapies by reducing cellular loss and enabling precise wound-specific deposition. As such, it has meaningful potential to enhance wound healing outcomes, streamline operative workflows, and expand the therapeutic options available for acute and chronic wounds within real-world burn and reconstructive surgical practice. Importantly, this approach represents a transformative step toward the future of soft-tissue reconstruction, where personalized, on-demand bioprinting will fundamentally change how complex tissue defects are repaired.

**Funding for the study:**

Institutional and sponsored funding.

